# Synthesis, Crystal Structure, Spectroscopic Properties, and Visible-Light Photoresponse of Lead-Free Cs_2_PdCl_4_·H_2_O

**DOI:** 10.3390/molecules31183259

**Published:** 2026-09-14

**Authors:** Wenhuan Cao, Chen Wang, Yu Li, Jie Yin, Huawei Zhou

**Affiliations:** 1 School of Materials Science and Engineering, Liaocheng University, Liaocheng 252000, China; 13777844469@163.com; 2 School of Chemistry and Chemical Engineering, Liaocheng University, Liaocheng 252000, China; 15966385758@163.com (C.W.); liyu19861901035@163.com (Y.L.); hwzhou10@163.com (H.Z.)

**Keywords:** Cs_2_PdCl_4_·H_2_O, lead-free palladium halide, square-planar coordination, visible-light photoresponse

## Abstract

Lead-free palladium halides containing square-planar coordination units provide a distinctive platform for investigating light-matter interactions, yet their spectroscopic behavior and thin-film photoresponse remain largely unexplored. Herein, we report a systematic investigation of Cs_2_PdCl_4_·H_2_O, synthesized as both nanorods and single crystals, covering its structural, spectroscopic, electronic, and photoresponsive properties. Single-crystal X-ray diffraction confirms that the monohydrate crystallizes in the orthorhombic Cmcm space group with discrete square-planar [PdCl_4_]^2−^ units. Powder X-ray diffraction verifies that the nanorods adopt the same crystalline phase and retain their principal diffraction features after two months of ambient storage. X-ray photoelectron spectroscopy confirms the Pd(II) valence state, and optical measurements determine a direct bandgap of 2.32 eV. Temperature-dependent and time-resolved photoluminescence reveal a low-temperature emission blueshift, enhanced emission intensity, and prolonged carrier decay, attributable to suppressed non-radiative relaxation and contributions from multiple emissive states. Density functional theory calculations based on the experimentally determined monohydrate structure show that the band-edge states are dominated by Cl 3p and Pd 4d orbitals. Thermally deposited Cs_2_PdCl_4_·H_2_O-derived films deliver reproducible visible-light photocurrent switching across 397–564 nm and retain 71% of their initial response after 504 h under nitrogen. These findings establish Cs_2_PdCl_4_·H_2_O as a visible-light-responsive lead-free palladium halide and provide a basis for future exploration in optoelectronic sensing.

## 1. Introduction

Photoresponsive semiconductors convert incident light into electrical or chemical responses and are central to optical sensing, imaging, photocatalysis, and solar-energy conversion. Their performance is governed by crystal structure, electronic states, optical transitions, carrier relaxation, and structural stability. Conventional inorganic semiconductors, despite mature optoelectronic performance, face limitations such as complex processing, high cost, toxicity, or limited compositional flexibility [1,2,3,4,5,6,7], motivating the exploration of structurally diverse, lead-free photoactive semiconductors.

Metal-halide semiconductors are attractive for their strong light absorption, tunable electronic structures, and versatile preparation methods [8,9,10,11,12]. However, Pb toxicity and the limited long-term stability of many lead-halide perovskites remain major obstacles to practical applications [13,14,15]. Lead-free halides, including A_2_BB′X_6_ double perovskites, A_2_BX_6_ vacancy-ordered compounds, and other low-dimensional or molecular metal halides, have thus been explored as alternatives [16,17,18,19,20,21]. Most studies have focused on octahedrally coordinated metal-halide frameworks, whereas non-octahedral lead-free halides remain less investigated, especially regarding spectroscopic behavior, carrier relaxation, and thin-film photoresponse [22].

Palladium(II) halides, owing to the 4d8 configuration of Pd(II), commonly adopt square-planar [PdX_4_]^2−^ units, distinct from the octahedral frameworks typical of conventional perovskites, thereby offering an opportunity to examine how non-octahedral coordination influences optical excitation and photoresponse. Cs_2_PdCl_4_ is an A_2_BX_4_-type molecular crystal built from discrete square-planar [PdCl_4_]^2−^ units, rather than a perovskite-type framework. Related compounds include Cs_2_PdBr_6_, a lead-free perovskite-type photoresponsive material. Previous studies have reported the preparation, vibrational properties, crystal growth, and room-temperature orthorhombic Cmcm phase of Cs_2_PdCl_4_, along with a tetragonal transition near 341 K and an anhydrous monoclinic polymorph that transforms to an orthorhombic hydrated phase under atmospheric moisture [23,24,25,26,27]. These results indicate that the phase behavior of cesium palladium chlorides is sensitive to hydration and preparation conditions. Within this family, our previous study demonstrated the photoresponsive behavior of square-planar Cs_2_PdBr_4_ [28]. Nevertheless, the spectroscopic properties and photoresponse of Cs_2_PdCl_4_·H_2_O, particularly in nanorod and thin-film forms, remain poorly understood.

Herein, we systematically investigate Cs_2_PdCl_4_·H_2_O in nanorod and single-crystal forms, focusing on its coordination structure, spectroscopic properties, electronic structure, and thin-film photoresponse. For the first time, we report temperature-dependent and time-resolved photoluminescence of this monohydrate, revealing emissive-state evolution and carrier relaxation pathways upon cooling. Density functional theory calculations based on the experimental structure clarify the orbital contributions to the band-edge states. Thermally deposited Cs_2_PdCl_4_·H_2_O-derived films exhibit reproducible visible-light photocurrent switching, providing the first thin-film photoresponse evaluation. These results establish Cs_2_PdCl_4_·H_2_O as a visible-light-responsive lead-free palladium halide with potential for optoelectronic sensing.

## 2. Results and Discussion

### 2.1. Synthesis, Morphology, Coordination Structure, and Phase Stability

**Figure 1:**
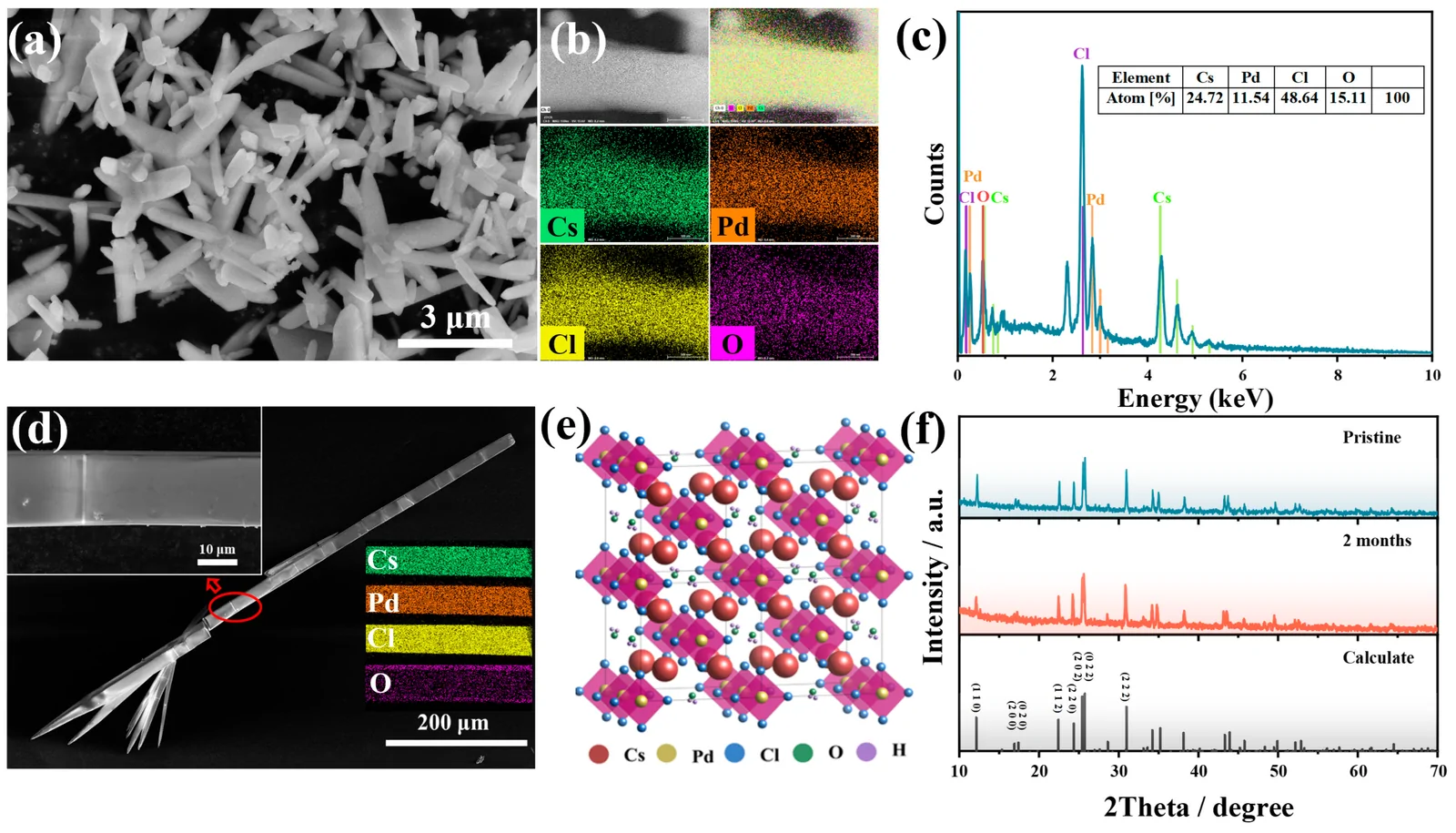
Morphological and structural characterization of Cs_2_PdCl_4_·H_2_O nanorods and single crystals: (**a**) SEM image of Cs_2_PdCl_4_·H_2_O nanorods; (**b**) elemental mapping of Cs, Pd, Cl and O; (**c**) EDS spectrum and elemental composition; (**d**) SEM image of a Cs_2_PdCl_4_·H_2_O single crystal and elemental mapping of Cs, Pd, Cl and O; (**e**) Crystal structure of Cs_2_PdCl_4_·H_2_O determined by SC-XRD; and (**f**) PXRD patterns of the Cs_2_PdCl_4_·H_2_O nanorods before and after two months of storage, together with the simulated pattern.

Cs_2_PdCl_4_·H_2_O nanorods were synthesized by a simple solution-precipitation method involving dissolution of CsCl and PdCl_2_ in concentrated HCl followed by IPA-induced precipitation, as illustrated in Figure S1. The scanning electron microscopy (SEM) image in Figure 1a shows that the as-prepared monohydrate predominantly exhibits a nanorod morphology. Energy-dispersive X-ray spectroscopy (EDS) mapping confirms homogeneous distributions of Cs, Pd, Cl, and O throughout the nanorods (Figure 1b). The Cs, Pd, Cl, and O atomic percentages are 24.72%, 11.54%, 48.64%, and 15.11%, respectively; after excluding O, the Cs, Pd, and Cl atomic percentages are 28.71%, 14.14%, and 57.19%, respectively, giving a Cs:Pd:Cl ratio of approximately 2:1:4 (Figure 1c). The O content exceeds the theoretical value for one crystalline water per formula unit (~3.5 at.%), attributable to the limited accuracy of EDS for light elements and possible contributions from surface-adsorbed moisture or trace oxygen-containing species.

Needle-like single crystals suitable for single-crystal X-ray diffraction (SC-XRD) were grown by liquid-phase diffusion from a separate synthesis batch (Figure 1d), enabling independent cross-validation of the crystalline phase. SC-XRD reveals that Cs_2_PdCl_4_·H_2_O crystallizes in the orthorhombic Cmcm space group (No. 63). Each Pd center is coordinated by four Cl atoms to form a square-planar [PdCl_4_]^2−^ unit with a Pd-Cl bond length of approximately 2.3 Å (Figure 1e). Water molecules connect adjacent [PdCl_4_]^2−^ units via O-H···Cl hydrogen bonds. This hydrogen-bonding network likely contributes to the stabilization of the orthorhombic Cmcm structure, as anhydrous Cs_2_PdCl_4_ adopts a distinctly different monoclinic C2/m structure [23]. The O-H···Cl interactions involve the chloride ligands directly coordinated to Pd(II), which may modulate the ligand field strength and thereby affect the d-orbital splitting and optical properties of the material. Detailed crystallographic parameters are summarized in Tables S1–S4. The isolated square-planar [PdCl_4_]^2−^ units distinguish Cs_2_PdCl_4_·H_2_O from conventional octahedral metal-halide frameworks and underpin its characteristic electronic and spectroscopic properties.

The experimental powder X-ray diffraction (PXRD) pattern of the nanorods matches that simulated from the single-crystal data (Figure 1f), confirming that both forms adopt the same orthorhombic monohydrate phase. This assignment is further supported by the distinct anhydrous phase, which crystallizes in the monoclinic C2/m space group [23,28], rather than the orthorhombic Cmcm structure determined here.

### 2.2. Surface Chemical States and Spectroscopic Properties

**Figure 2:**
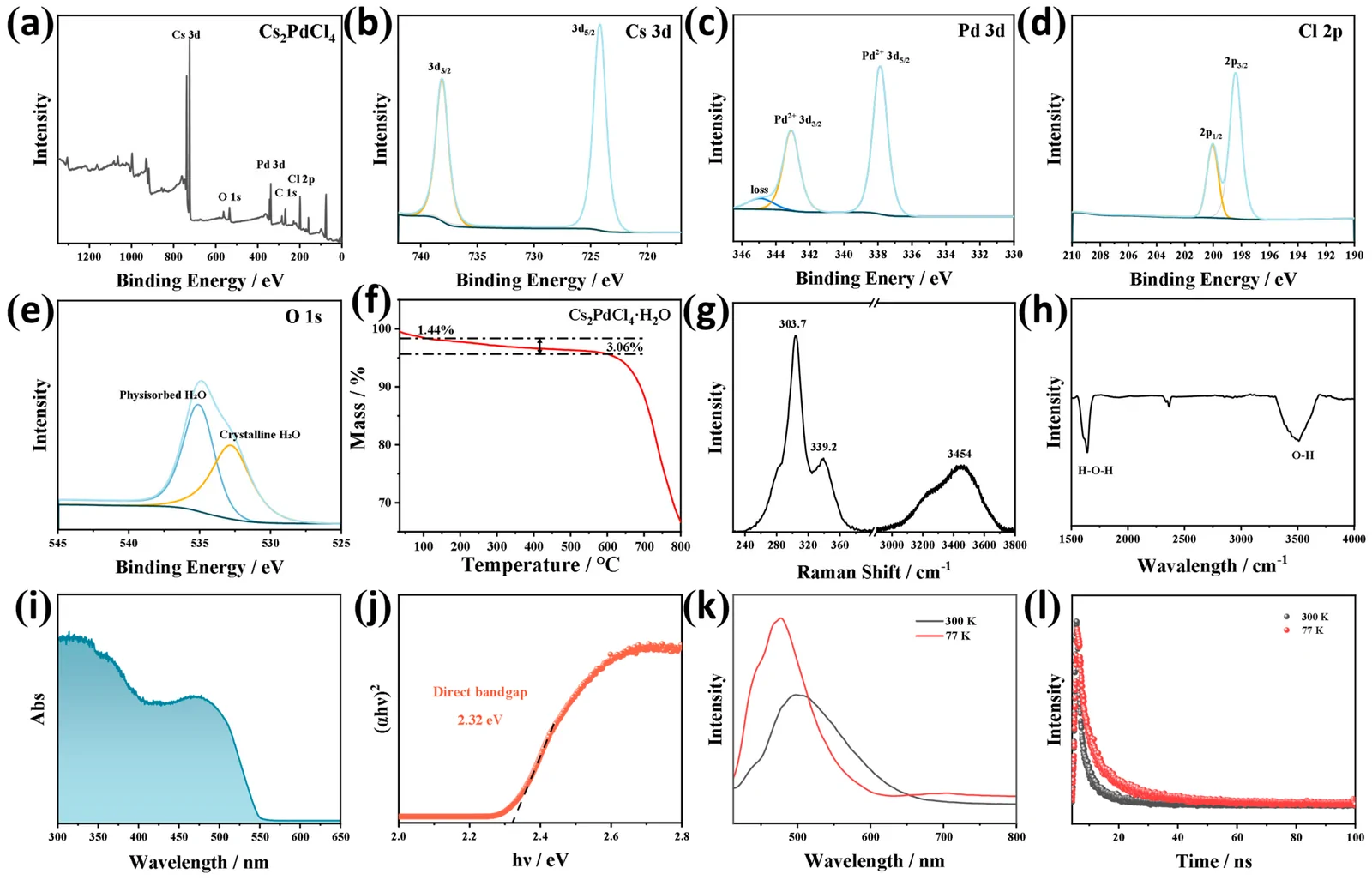
XPS spectra of (**a**) Full spectrum (**b**) Cs 3d, (**c**) Pd 3d, (**d**) Cl 2p, and (**e**) O 1s; (**f**) TGA curve of Cs_2_PdCl_4_·H_2_O measured under N_2_ atmosphere; (**g**) Raman spectrum; (**h**) FTIR spectrum; (**i**) UV-vis absorption spectrum; (**j**) Tauc plot; (**k**) temperature-dependent PL spectra; and (**l**) TRPL decay curves.

The surface chemical states of the Cs_2_PdCl_4_·H_2_O nanorods were characterized by X-ray photoelectron spectroscopy (XPS) (Figure 2a–e). In the high-resolution Cs 3d spectrum, the peaks at 724.0 and 737.95 eV are assigned to Cs 3d_5/2_ and Cs 3d_3/2_, respectively [29,30]. The Pd 3d spectrum shows peaks at 337.85 and 343.10 eV, corresponding to Pd 3d_5/2_ and Pd 3d_3/2_, consistent with the Pd(II) oxidation state [31,32]. The Cl 2p_3/2_ and Cl 2p_1/2_ peaks are located at 198.2 and 199.8 eV, respectively [33]. The O 1s spectrum can be deconvoluted into two components: a peak at 532.85 eV attributed to crystalline water in the structure, and a peak at 535.1 eV assigned to physisorbed water on the surface [34]. The presence of these two distinct O 1s components confirms the existence of both crystalline water and surface-adsorbed water in the sample. This result is in good agreement with the TGA and SC-XRD conclusions, further corroborating the monohydrate structure of Cs_2_PdCl_4_·H_2_O from the surface chemistry perspective.

To further confirm that the water in the nanorods is crystalline rather than adsorbed, thermogravimetric analysis (TGA) was performed (Figure 2f). The TGA curve shows two mass-loss stages: 1.44% below 100 °C, assigned to surface-adsorbed water, and 3.06% up to 600 °C, assigned to crystalline water. The latter is close to the theoretical value of 3.39% for stoichiometric Cs_2_PdCl_4_·H_2_O; the slight deviation may arise from partial loss of crystalline water during desorption of adsorbed water. Because all samples were dried at 100 °C before characterization, these results confirm that the drying step removes only adsorbed water while preserving crystalline water. Principal diffraction peaks remained essentially unchanged after two months under ambient conditions, indicating retention of the Cs_2_PdCl_4_·H_2_O phase under the tested conditions.

The presence of water in Cs_2_PdCl_4_·H_2_O was further confirmed by Raman and Fourier-transform infrared (FTIR) spectroscopies. As shown in Figure 2g, the Raman spectrum exhibits two sharp peaks at 303.7 and 339.2 cm^−1^, which are characteristic of Pd-Cl stretching vibrations within the square-planar [PdCl_4_]^2−^ units, consistent with the coordination environment determined by SC-XRD. In addition, a broad band centered at 3454 cm^−1^ is observed, which is characteristic of O-H stretching vibrations of water molecules. The FTIR spectrum (Figure 2h) shows absorption bands at approximately 3450 and 1630 cm^−1^, corresponding to O-H stretching and H-O-H bending vibrations, respectively, further confirming the presence of water in the structure.

The optical absorption spectrum (Figure 2i) and corresponding Tauc plot (Figure 2j) yield a direct allowed optical bandgap of 2.32 eV. Temperature-dependent photoluminescence spectra (Figure 2k) reveal pronounced changes in emission position, linewidth, and intensity. At 300 K, the emission consists of a broad asymmetric band centered near 500 nm with a weak high-energy shoulder; upon cooling to 77 K, it shifts to approximately 470 nm, narrows, and increases markedly in intensity. These changes are consistent with reduced electron-phonon coupling and suppressed thermally activated non-radiative relaxation at low temperature [35,36,37]. The asymmetric line shape further suggests contributions from multiple emissive states.

Time-resolved photoluminescence decay at 77 K is slower than at 300 K (Figure 2l, Table S5), indicating a longer effective carrier lifetime at low temperature. Biexponential fitting resolves fast and slow decay components, corresponding to rapid and long-lived relaxation channels. The larger slow-component contribution at 77 K is consistent with reduced non-radiative loss and enhanced radiative relaxation [38,39]. Overall, cooling redistributes emissive states and steers carrier relaxation toward longer-lived pathways.

### 2.3. Electronic Structure and Band-Edge Characteristics

**Figure 3:**
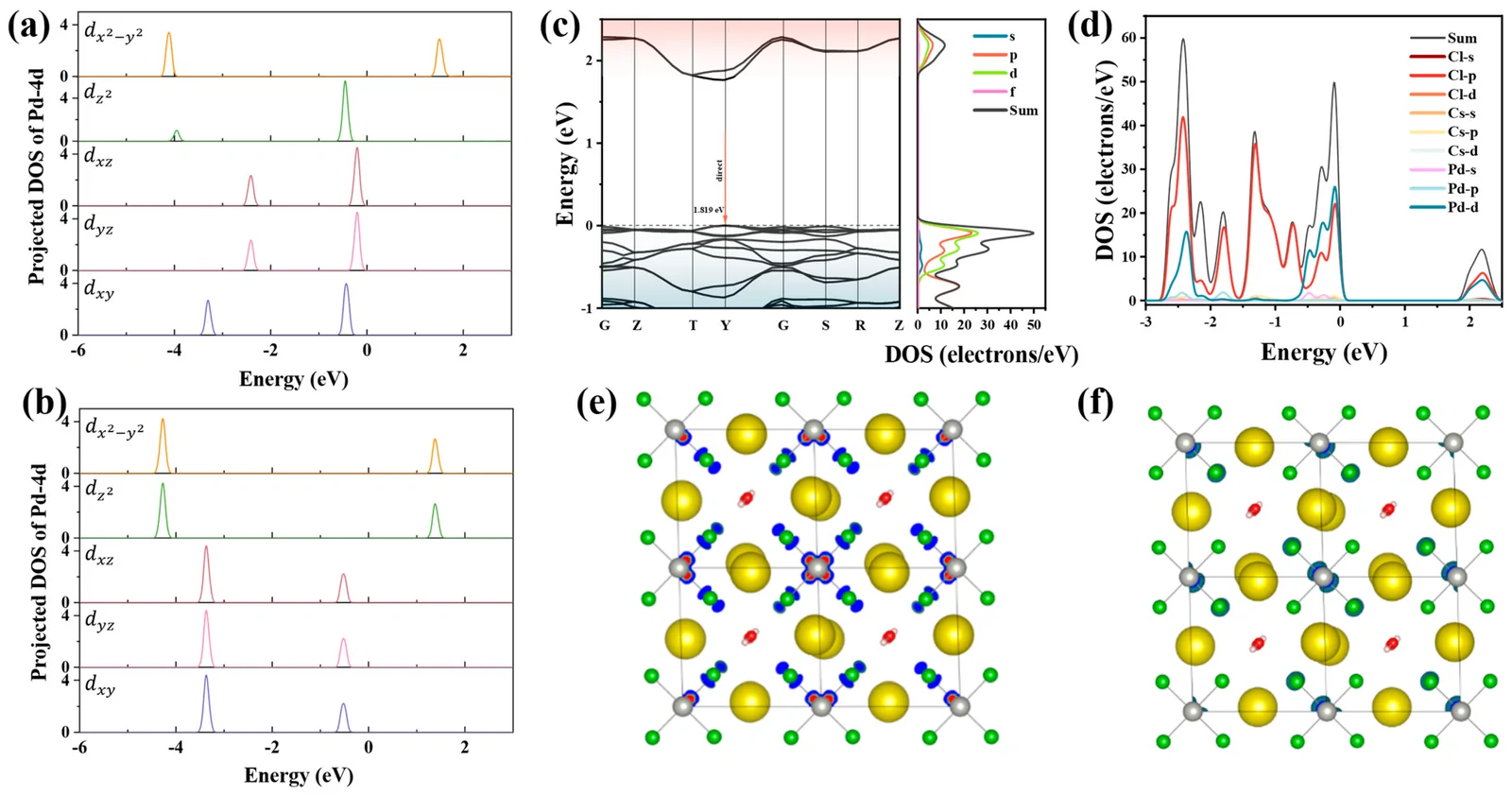
Partial density of states (DOS) calculations of Pd center: (**a**) PdCl_4_^2−^; (**b**) PdCl_6_^2−^; Calculated electronic structure of Cs_2_PdCl_4_·H_2_O: (**c**) band structure and total density of states; (**d**) projected density of states; and band-decomposed charge-density distributions corresponding to the (**e**) CBM and (**f**) VBM.

To clarify the electronic consequence of the square-planar coordination, the Pd 4d states were further resolved into individual orbital components and compared with those of an octahedral reference model (Figure 3a,b) [40]. In the octahedral environment, the $d_{xy}$, $d_{xz}$, and $d_{yz}$ states exhibit nearly identical distributions, while the $d_{x^{2}-y^{2}}$ and $d_{z^{2}}$ components also show closely similar features, consistent with the conventional $t_{2g}/e_{g}$ crystal-field splitting. In contrast, removal of the two axial ligands in the square-planar structure further lifts the orbital degeneracy. The $d_{xz}$ and $d_{yz}$ states remain approximately degenerate, whereas the $d_{xy}$, $d_{z^{2}}$, and $d_{x^{2}-y^{2}}\;$components become clearly separated.

In particular, the square-planar structure exhibits a pronounced unoccupied $d_{x^{2}-y^{2}}$-derived state. Because this orbital points directly toward the four in-plane Cl ligands, it is strongly destabilized by Pd-Cl σ-antibonding interactions. Combined with the strong Cl-3p contribution at the valence-band edge and Pd-4d contribution at the conduction-band edge, this result indicates that the band-edge excitation has substantial ligand-to-metal charge-transfer character. Thus, the square-planar ligand field directly reorganizes the Pd 4d manifold and shapes the electronic states responsible for visible-light excitation.

Having established how the square-planar ligand field reorganizes the Pd 4d manifold, we next examined how this orbital redistribution is reflected in the band-edge electronic states and optical excitation. The calculated band structure (Figure 3c) shows that Cs_2_PdCl_4_·H_2_O is a direct-bandgap semiconductor with a bandgap of 1.819 eV at the Y point. This value is smaller than the experimentally estimated optical gap of 2.32 eV, consistent with the well-known tendency of the PBE functional to underestimate semiconductor bandgaps [41]. The orbital-resolved PDOS (Figure 3d) further links the crystal-field splitting to the band-edge composition. The valence-band maximum is dominated by Cl 3p states, whereas the conduction-band minimum contains substantial Pd 4d character, including the strongly destabilized $d_{x^{2}-y^{2}}\;$-derived state identified from the square-planar crystal-field analysis [42]. The band-decomposed charge densities (Figure 3e,f and Figure S2) further show that both band-edge states are primarily localized within the Pd-Cl framework. These results indicate that the band-edge excitation involves electron transfer from predominantly Cl-derived occupied states to Pd-derived unoccupied states and therefore has substantial ligand-to-metal charge-transfer character, rather than corresponding to a simple pure metal-centered d-d transition. Thus, the square-planar ligand field not only lifts the Pd 4d orbital degeneracy but also directly shapes the orbital character of the states involved in visible-light excitation, providing an electronic-structure basis for the observed optical absorption and photocurrent response.

### 2.4. Visible-Light Photoresponse of Thermally Deposited Films

**Figure 4:**
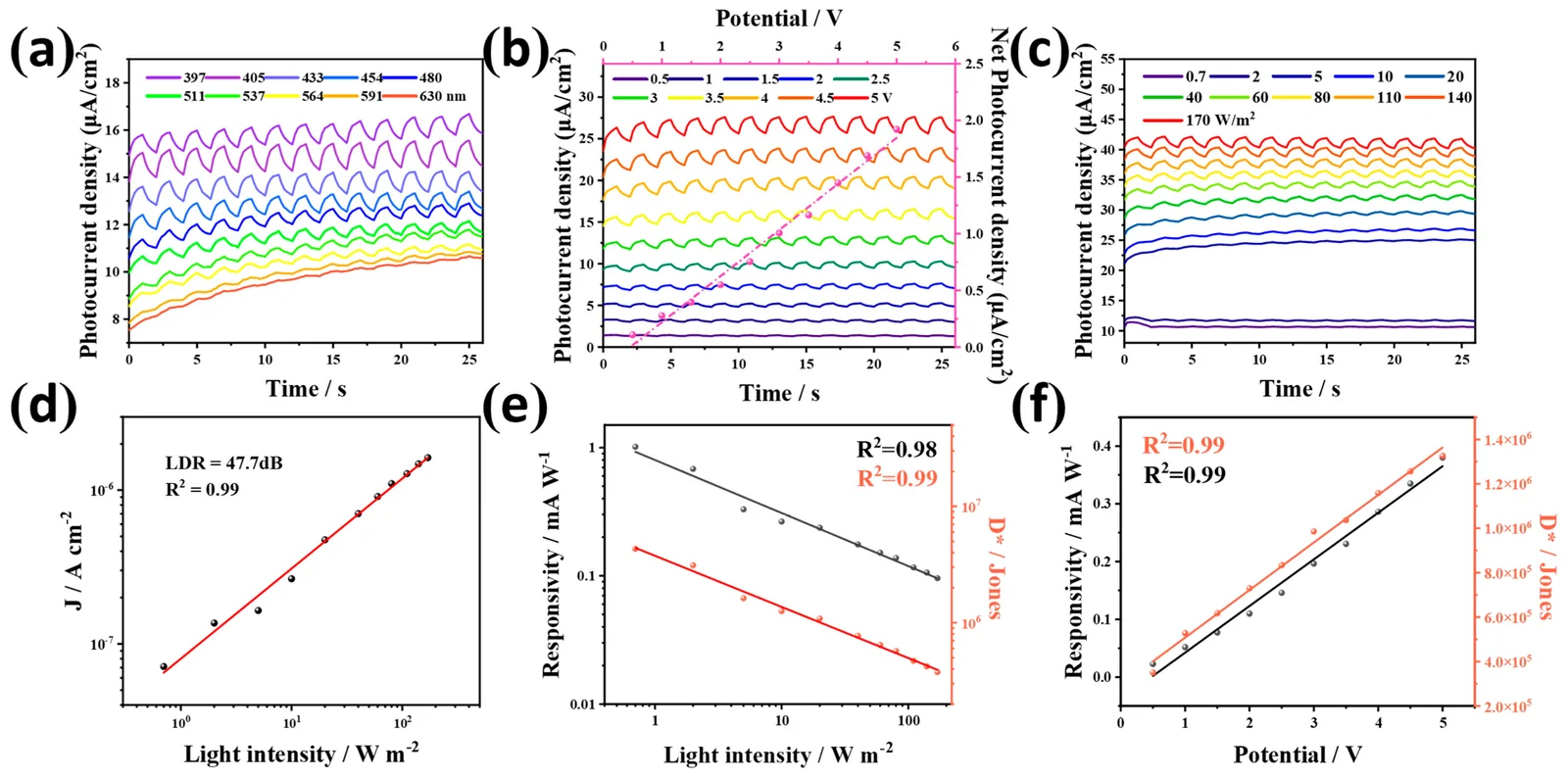
Visible-light photoresponse characteristics of the thermally deposited Cs_2_PdCl_4_·H_2_O-derived film: (**a**) J-t curves under different wavelengths of light at 5 V bias voltage; (**b**) J-t curves at 397 nm and fitted net photocurrent densities at different bias voltages; (**c**) J-t curves at 397 nm under different light intensities; (**d**) LDR; (**e**) Responsivity and specific detectivity as a function of light intensity; (**f**) Responsivity and specific detectivity as a function of voltage.

To evaluate the intrinsic visible-light photocurrent-generation capability of Cs_2_PdCl_4_·H_2_O without heterojunction or interfacial engineering, a Cs_2_PdCl_4_·H_2_O-derived layer was thermally deposited onto ITO interdigital electrodes. The effective illuminated area was 0.04 cm^2^, and the measurement configuration is shown in Figure S3. SEM and elemental mapping confirm the formation of a continuous film containing Cs, Pd, and Cl with a relatively homogeneous elemental distribution (Figure S4). Current-voltage curves measured in the dark and under 405 nm irradiation show a gradual increase in current with increasing light intensity (Figure S5), consistent with photogenerated carrier collection. Figure 4a presents the wavelength-dependent current-time response at an incident power density of 50 W m^−2^ and a bias voltage of 5 V. Reproducible switching responses were observed between 397 and 564 nm; weak signals at 591 and 630 nm lacked sufficient periodicity for inclusion in the effective response range. The decrease in photocurrent toward longer wavelengths is consistent with the optical absorption behavior, demonstrating that the thermally deposited film can generate and transport photocarriers under visible-light excitation.

Figure 4b shows current-density-time curves measured at different bias voltages. The net photocurrent density increases approximately linearly with bias voltage from 0.5 to 5 V (R^2^ = 0.99), indicating that the applied electric field promotes carrier separation and collection. However, higher bias may also increase dark current, noise, power consumption, and electrical stress; therefore, the photocurrent increase alone should not be interpreted as an unconditional improvement in device performance [43].

The photocurrent dependence on incident light intensity is shown in Figure 4c. A reproducible response remains detectable down to 0.7 W m^−2^, and the photocurrent density increases progressively with light intensity. Responsivity (*R*), shot-noise-limited specific detectivity (D*), and linear dynamic range (*LDR*) were calculated as quantitative descriptors of the film-level photoresponse. *LDR* was calculated as [44]

(1) $$LDR=20log\frac{J_{upper}}{J_{lower}}=20log\frac{P_{upper}}{P_{lower}}$$

where *J_upper_* and *J_lower_* are the upper and lower photocurrent-density limits, and *P_upper_* and *P_lower_* are the corresponding incident light intensities; the resulting *LDR* is 47.7 dB (Figure 4d). R was calculated as

(2) $$R=\frac{I_{PC}-I_{Dark}}{P_{in}\times s}$$

where *I_PC_*, *I_dark_*, *P_in_*, and *S* denote the photocurrent, dark current, incident light power density, and effective illuminated area, respectively. D* was estimated as

(3) $$D^{*}=\frac{R}{\sqrt{2eJ_{Dark}}}$$

where e is the elementary charge and *J_dark_* is the dark-current density. Because this expression assumes dark-current shot-noise-limited operation, the result is reported as the shot-noise-limited specific detectivity rather than as a value from direct noise measurements [45].

As shown in Figure 4e, both *R* and D* decrease with increasing incident light intensity, likely due to trap-state filling and enhanced carrier recombination at higher photocarrier densities [46]. Under the tested conditions, the device achieves a maximum *R* of 1.02 mA/W and D* of 4.31 × 10^6^ Jones at 397 nm, 0.7 W m^−2^, and 5 V. Although these values remain below those of several optimized lead-free halide photodetectors, the simple ITO/Cs_2_PdCl_4_·H_2_O-derived architecture demonstrates that the material can be processed into a photoresponsive thin film without additional heterojunction or interfacial engineering.

*R* and D* both increase approximately linearly with bias voltage (R^2^ = 0.99, Figure 4f), consistent with improved carrier separation and collection. The Cs_2_PdCl_4_·H_2_O-derived film exhibits larger *R* and D* than those previously measured for the related Cs_2_PdBr_4_-derived film. Because the two systems differ in halide composition, hydration state, film microstructure, and measurement conditions, this comparison should be regarded as evidence of the functional diversity of cesium palladium halides rather than as proof of a solely chloride-induced enhancement. Selected photoresponse parameters are compared with those of related lead-free halide devices in Table 1; given substantial differences in device architecture and measurement conditions, the comparison is qualitative and is not intended to establish performance superiority. The transient response in Figure S6 gives rise and decay times of 290 and 393 ms, respectively, defined as the time for the photocurrent to change between 10% and 90% of its steady-state value.

The visible-light absorption and reproducible photocurrent generation confirm the photoactive nature of Cs_2_PdCl_4_·H_2_O. Its ability to generate and transport photoexcited carriers provides a basis for broader light-driven applications, including optical sensing and photocatalysis, such as organic pollutant degradation or hydrogen evolution [47]. Systematic evaluation of its photocatalytic performance remains a subject for future work.

**Table 1:** Selected quantitative photoresponse parameters of the Cs_2_PdCl_4_·H_2_O-derived film and related lead-free halide devices.

Device	Responsivity (mA/W)	Detectivity (Jones)	LDR (dB)	Ref.
ITO/Cs_2_PdCl_4_·H_2_O	1.02	4.31 × 10^6^	47.7	This work
ITO/Cs_2_PdBr_4_	0.27	1.13 × 10^6^	24.08	[28]
ITO/Cs_2_SnI_6_	1.07	10^10^		[48]
ITO/Cs_2_SnCl_6_/Pt	67.8	4.17 × 10^11^	40	[49]
ITO/Rb_2_TeI_6_	1.4	10^10^		[50]

### 2.5. Photoresponse Reproducibility and Storage Stability

**Figure 5:**
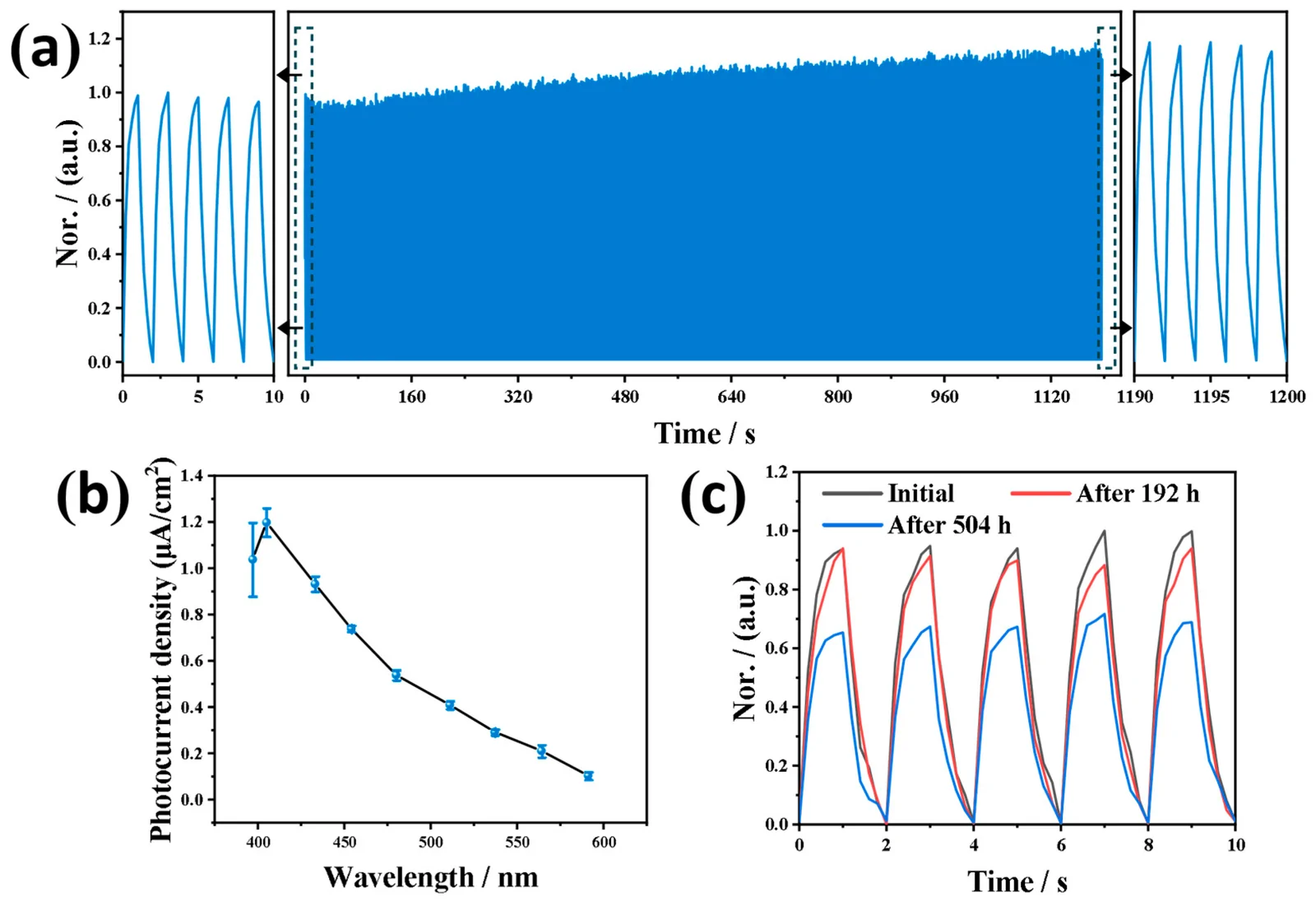
Cyclic photoresponse, sample-to-sample reproducibility, and storage retention of Cs_2_PdCl_4_·H_2_O-derived films: (**a**) photocurrent switching during a 20 min illumination test; (**b**) variation in photocurrent density at different wavelengths; and (**c**) photoresponse retention after storage under nitrogen.

The short-term photoresponse of the Cs_2_PdCl_4_·H_2_O-derived film was evaluated under 405 nm irradiation at 4 V and 170 W m^−2^. As shown in Figure 5a, the film exhibits stable, periodic photocurrent switching over 20 min, while the amplitude gradually increases to approximately 125% of its initial value before stabilizing. This gradual enhancement is ascribed to trap filling rather than material degradation or device instability. The biexponential carrier decay revealed by time-resolved PL measurements confirms the presence of intrinsic trap states. Upon initial illumination, photogenerated carriers progressively saturate these traps, suppressing non-radiative recombination and improving charge collection efficiency [51,52].

Device-to-device reproducibility was evaluated for samples from the same batch at different wavelengths (Figure 5b). The devices exhibit comparable wavelength-dependent response trends, with the strongest response near 405 nm. This analysis primarily reflects fabrication reproducibility rather than temporal operating stability.

The storage stability of the film-level photoresponse was evaluated by storing samples in an N_2_-filled glovebox. After 504 h, approximately 71% of the initial photoresponse was retained (Figure 5c). Together with the essentially unchanged powder XRD pattern after two months, these results indicate that Cs_2_PdCl_4_·H_2_O retains its principal structural and photoresponsive characteristics under the respective storage conditions.

## 3. Materials and Methods

Materials: CsCl (Cesium chloride, 99%; J&K Scientific Ltd., Beijing, China), PdCl_2_ (Palladium chloride, 98%; Vetec, St. Louis, MO, USA), HCl (Hydrochloric acid, 36–38 wt% in water; Yantai Far East Fine Chemical Co., Ltd., Yantai, Shandong, China), DMSO (Dimethyl sulfoxide, A.R.; Shanghai Macklin Biochemical Co., Ltd., Shanghai, China), IPA (Isopropyl alcohol, A.R., ≥99.5%; Shanghai Macklin Biochemical Co., Ltd., Shanghai, China). All reagents were used without further purification.

Physical Measurements: SEM images and EDS mapping were obtained using a Thermo Fisher Scientific FIB-SEM GX4 electron microscope (Thermo Fisher Scientific, Waltham, MA, USA) operated at 10 kV and 86 pA. Single-crystal X-ray diffraction data were collected on a Bruker D8 Venture diffractometer (Bruker AXS GmbH, Karlsruhe, Germany). Powder X-ray diffraction patterns were collected on a Rigaku Smartlab X-ray diffractometer (Rigaku Corporation, Tokyo, Japan). UV-Vis absorption and diffuse reflectance spectra were recorded on a PerkinElmer UV-Vis-NIR spectrometer (PerkinElmer, Inc., Waltham, MA, USA) with BaSO_4_ as the reflectance reference. Photocurrent-time measurements were performed using a Zahner CIMPS-2 pro electrochemical workstation (Zahner-Elektrik GmbH & Co. KG, Kronach, Germany). Photoluminescence spectra were recorded on an FLS1000 fluorescence spectrometer (Edinburgh Instruments Ltd., Livingston, UK). XPS measurements were performed on a Thermo Scientific Escalab Xi+ X-ray photoelectron spectrometer (Thermo Fisher Scientific, Waltham, MA, USA) equipped with an Al Kα excitation source. All spectra were collected with an X-ray spot size of 500 μm. Survey spectra were acquired in constant analyzer energy mode at a pass energy of 100.0 eV, an energy step of 1.000 eV, and 2 scan cycles. High-resolution core-level spectra (Cs 3d, Pd 3d, Cl 2p, O 1s, C 1s) were acquired at a pass energy of 30.0 eV, an energy step of 0.050 eV, and 5 scan cycles. TGA was performed on a NETZSCH STA 449F5 (NETZSCH-Gerätebau GmbH, Selb, Germany) under N_2_ at a heating rate of 10 K min^−1^ from room temperature to 1273 K. FTIR spectra were recorded using an INVENIO FTIR spectrometer (Bruker Optik GmbH, Ettlingen, Germany). Raman spectra were collected at room temperature using a Renishaw inVia Raman spectrometer (Renishaw plc, Wotton-under-Edge, UK) equipped with a 532 nm excitation laser over a Raman-shift range of 400–4000 cm^−1^.

Theoretical calculations Methods: To analyze the d-orbital splitting and partial density of states (PDOS) of the transition-metal center, plane-wave-based periodic DFT calculations were performed using the Vienna ab initio simulation package (VASP) [53,54]. The ionic cores were described by the projector augmented wave (PAW) method [55,56], and the exchange-correlation interactions were treated with the PBE functional within the generalized gradient approximation (GGA). To achieve accurate energies with errors of less than 1 meV per atom, the cut-off energy for plane-wave expansion was set to 520 eV, and the convergence criteria for forces were set to below 0.02 eV/Å, with an iterative convergence of energy of 10^−6^ eV. To analyze the band structure, DOS, and charge density distribution of Cs_2_PdCl_4_·H_2_O, DFT calculations were performed using the CASTEP module implemented in the Materials Studio 8.0 software package [57]. The exchange–correlation functional was treated by the GGA with the PBE functional [40]. A plane-wave cutoff energy of 571.4 eV was employed for all calculations. The convergence criteria for geometry optimization were set as follows: energy change less than 10^−5^ eV per atom, maximum force less than 0.03 eV Å^−1^, maximum displacement less than 0.001 Å, and maximum stress less than 0.05 GPa. The structural model was built directly from the experimentally determined single-crystal structure of Cs_2_PdCl_4_·H_2_O obtained in this work, with the crystallographic water molecule retained in the calculation. For the electronic structure calculations, the properties, including band structure and DOS, were calculated after full geometry optimization.

Synthesis of Cs_2_PdCl_4_·H_2_O nanorods: CsCl (0.6734 g) and PdCl_2_ (0.3546 g) were dissolved in HCl (10 mL, 36–38 wt%) at 85 °C for 5 min, followed by further heating to 120 °C until a clear solution formed. After cooling to room temperature, excess IPA was added to precipitate a large amount of solid. The precipitate was collected by centrifugation (7000 rpm, 3 min) and washed with IPA five times, then dried at 100 °C for 1 h to obtain Cs_2_PdCl_4_·H_2_O nanorods.

Synthesis of Cs_2_PdCl_4_·H_2_O Single crystals: Single crystals were prepared by liquid-phase diffusion. In a 20 mL vial, Cs_2_PdCl_4_·H_2_O nanorods (25 mg) were dissolved in HCl (1.5 mL) to form a clear solution. Additional HCl (1.5 mL) was then slowly added as a buffer layer, followed by slow addition of IPA (17 mL). After standing for 3 days, the product was collected by filtration, washed with IPA, and dried at 100 °C for 1 h to yield needle-like Cs_2_PdCl_4_·H_2_O single crystals.

Preparation of Films for Photoresponse Measurements: ITO interdigital electrode substrates were cleaned sequentially by ultrasonication in detergent, distilled water, and ethanol. Thin films were prepared by thermal evaporation of 100 mg of Cs_2_PdCl_4_·H_2_O nanorods at a deposition rate of 0.2 Å s^−1^, under 3 × 10^−5^ Pa.

## 4. Conclusions

In summary, Cs_2_PdCl_4_·H_2_O was synthesized as nanorods and single crystals and systematically characterized. Single-crystal X-ray diffraction established an orthorhombic Cmcm structure built from square-planar [PdCl_4_]^2−^ units, while PXRD confirmed the same monohydrate phase for the nanorods. XPS supports the Pd(II) assignment, and optical measurements yield a direct bandgap of 2.32 eV. Temperature-dependent and time-resolved photoluminescence reveal low-temperature spectral narrowing, enhanced emission, and slower carrier decay, consistent with reduced non-radiative relaxation and multiple emissive pathways. Density functional theory shows that Cl 3p and Pd 4d orbitals dominate the band-edge states. Thermally deposited Cs_2_PdCl_4_·H_2_O-derived films exhibit reproducible photocurrent switching over 397–564 nm. These results provide a comprehensive structural, spectroscopic, electronic, and photoresponsive description of Cs_2_PdCl_4_·H_2_O. The visible-light absorption and film-level photocurrent response highlight its potential for optical sensing and broader light-driven applications, including photocatalysis.

## Data Availability

The original contributions presented in this study are included in the article. Further inquiries can be directed to the corresponding author.

## Supplementary Materials

The following supporting information can be downloaded at https://www.mdpi.com/article/10.3390/molecules31183259/s1, Figure S1. Schematic illustration of the synthesis of Cs_2_PdCl_4_·H_2_O nanorods by a solution-precipitation method; Figure S2. (a) Partial PDOS of Pd; (b) complete PDOS of Pd; Figure S3. ITO interdigital electrodes evaporated with Cs_2_PdCl_4_; Figure S4. (a) SEM images of the surface morphology of Cs_2_PdCl_4_-TF deposited on ITO-IEs; element mapping images of Cs (b), Pd (c), and Cl (d) on ITO-IEs; (e) SEM images of the cross-section morphology of Cs_2_PdCl_4_-TF deposited on ITO-IEs; (f) Mapping test area and mapping image of each element of cross-sectioned Cs_2_PdCl_4_-TF; Figure S5. LSV images of Cs_2_PdCl_4_-TF in the dark and at different light intensities at 405 nm; Figure S6. Single response cycle of Cs_2_PdCl_4_·H_2_O-derived photoresponse device at 397 nm to determine response time; Table S1. Single X-ray diffraction crystallographic data of Cs_2_PdCl_4_·H_2_O SCs; Table S2. Atomic Coordinates and Equivalent Isotropic Displacement Parameters (Å^2^); Table S3. Anisotropic Displacement Parameters (Å^2^); Table S4. Selected Bond Lengths; Table S5. TRPL fitting parameters for room temperature and low temperature of Cs_2_PdCl_4_·H_2_O SCs.

## Abbreviations

The following abbreviations are used in this manuscript:

D*	detectivity
R	responsivity
DFT	density-functional theory
PBE	Perdew-Burke-Ernzerhof
SEM	scanning electron microscopy
EDS	Energy-dispersive X-ray spectroscopy
SC-XRD	Single-crystal X-ray diffraction
PL	photoluminescence
UV-vis	ultraviolet-visible
TRPL	time-resolved photoluminescence
LMCT	ligand-to-metal charge transfer
CBM	conduction band minimum
VBM	valence band maximum
TF	thin film
ITO-IEs	ITO interdigital electrodes
J-t	current density-time curves
LDR	linear dynamic range
PXRD	powder X-ray diffraction
TGA	thermogravimetric analysis
FTIR	Fourier-transform infrared
