# Supplementary material for: Synthesis, Crystal Structure, Spectroscopic Properties, and Visible-Light Photoresponse of Lead-Free Cs2PdCl4·H2O
Source: Molecules. 2026 Sep 14;31(18):3259. doi: 10.3390/molecules31183259 (PMC13609189; doi:10.3390/molecules31183259)
Supplement: Supplementary file 1 [file molecules-31-03259-s001.zip › SI.pdf]

Synthesis, Crystal Structure, Spectroscopic Properties, and Visible-Light Photoresponse of Lead-Free $Cs_2PdCl_4{\cdot}H_2O$

Wenhuan Cao [1], Chen Wang [2], Yu Li [2], Jie Yin [1,*] and Huawei Zhou [2]

[1] School of Materials Science and Engineering, Liaocheng University, Liaocheng 252000, China; 13777844469@163.com
[2] School of Chemistry and Chemical Engineering, Liaocheng University, Liaocheng 252000, China; 15966385758@163.com (C.W.); liyu19861901035@163.com (Y.L.); hwzhou10@163.com (H.Z.)
* Correspondence: yinjie@lcu.edu.cn

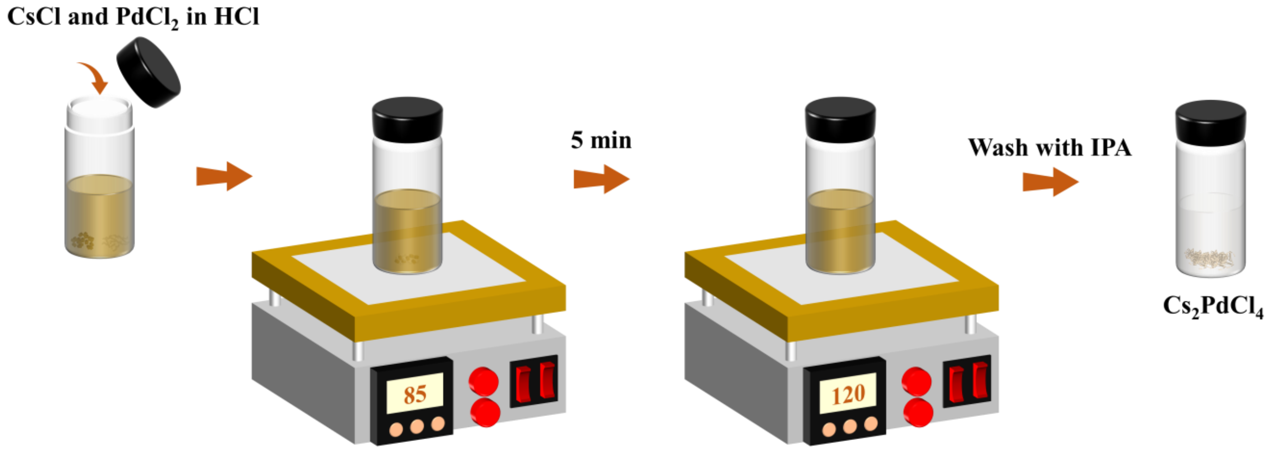

Figure S1. Schematic illustration of the synthesis of $Cs_2PdCl_4{\cdot}H_2O$ nanorods by a solution-precipitation method.

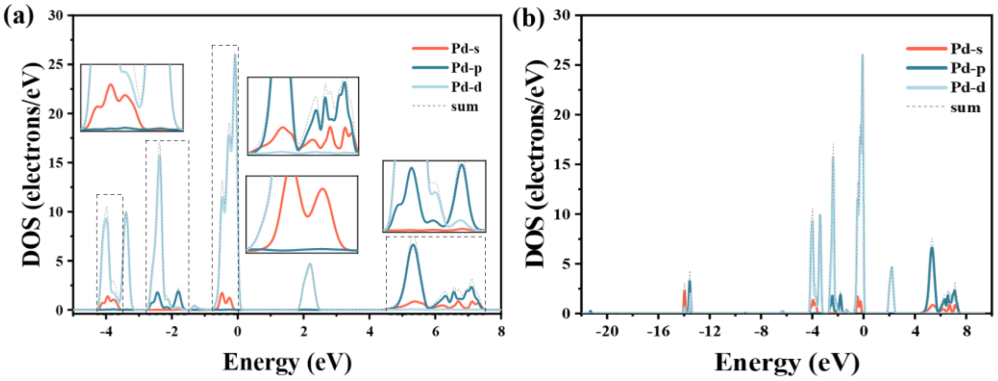

Figure S2. (**a**) Partial PDOS of Pd; (**b**) complete PDOS of Pd.

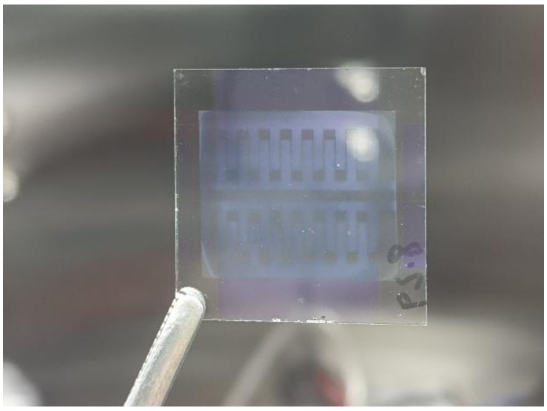

Figure S3. ITO interdigital electrodes evaporated with Cs_2PdCl_4.

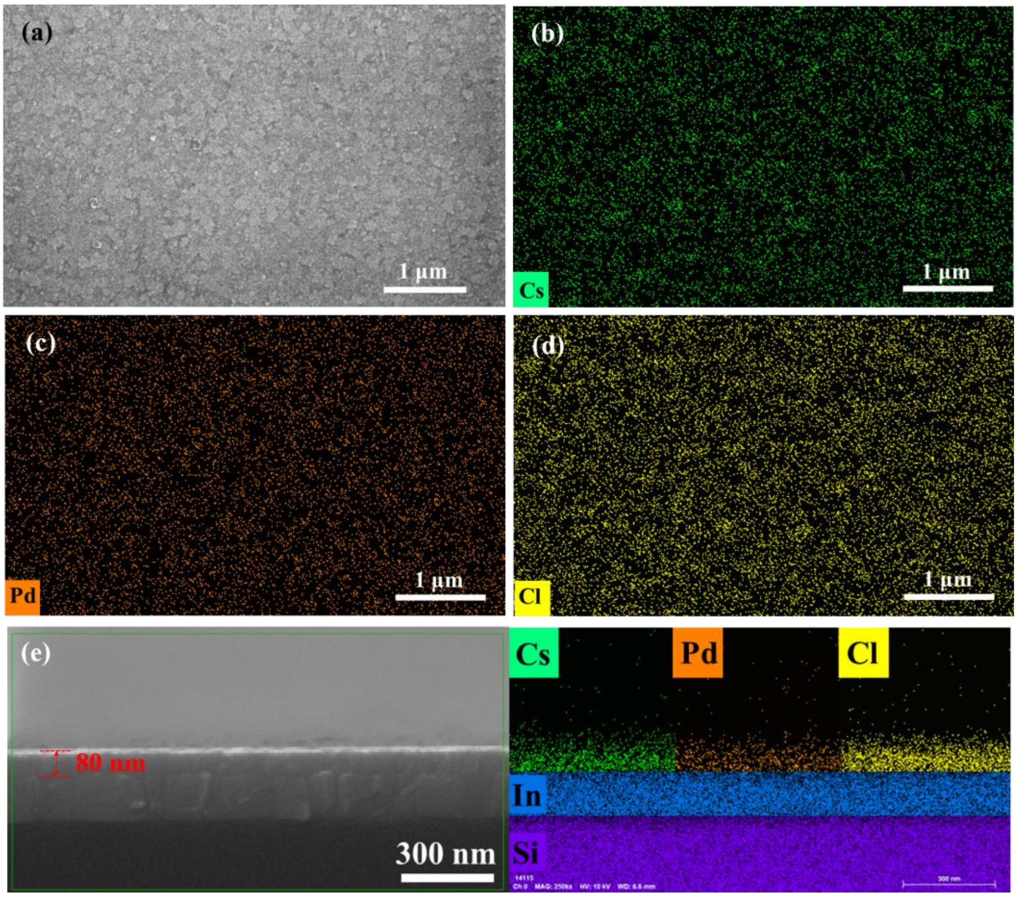

Figure S4. (**a**) SEM images of the surface morphology of Cs_2PdCl_4-TF deposited on ITO-IEs; element mapping images of Cs (**b**), Pd (**c**), and Cl (**d**) on ITO-IEs; (**e**) SEM images of the cross-section morphology of Cs_2PdCl_4-TF deposited on ITO-IEs; (**f**) Mapping test area and mapping image of each element of cross-sectioned Cs_2PdCl_4-TF.

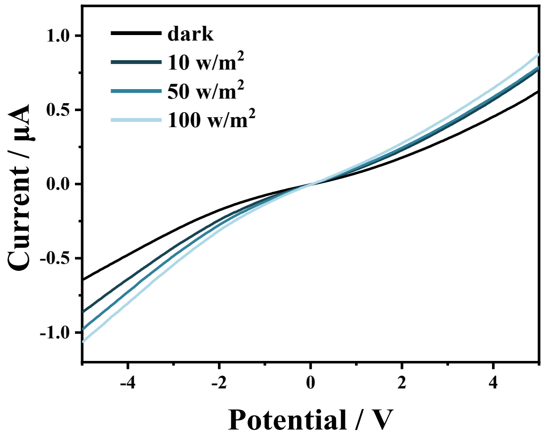

Figure S5. LSV images of Cs_2PdCl_4-TF in the dark and at different light intensities at 405 nm.

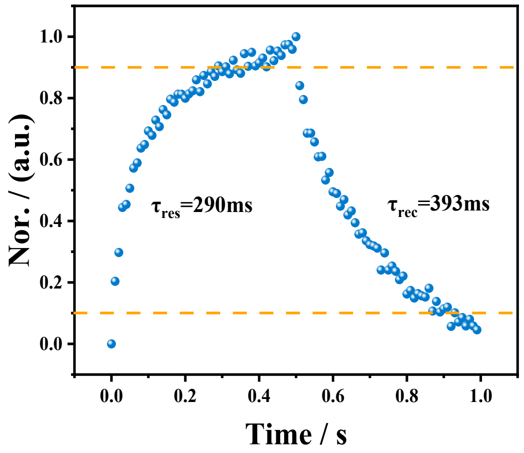

Figure S6. Single response cycle of $Cs_2PdCl_4{\cdot}H_2O$-derived photoresponse device at 397 nm to determine response time.

Table S1. Single X-ray diffraction crystallographic data of $Cs_2PdCl_4{\cdot}H_2O$ SCs.

**Empirical Formula**	**$Cs_{0.8}Pd_{0.4}Cl_{1.6}{\cdot}H_{0.8}O_{0.4}$**
Formula weight	212.81 g mol^{-1}
Temperature	223 K
Wavelength	0.71073 Å
Crystal system	Orthorhombic
Space group	*C m c m*
Unit cell dimensions	a = 10.4929(3) Å α = 90°<br>b = 10.2045(3) Å β = 90°<br>c = 9.4489(3) Å γ = 90°
Volume/Å^3	1011.74(5)
Z	4
Density	3.493 g cm^{-3}
Absorption coefficient/mm^{-1}	9.910
F(000)	936
Crystal description/color	Needle/Yellowish green
Radiation type/wavelength	Mo Kα, λ = 0.71073 Å
Monochromator	Graphite
Scan method	φ and ω scans
Reflections collected	8833
Independent reflections	650 [R(int) = 0.0491]
Completeness (θ = 25.242°)	99.6%
Absorption correction	Semi-empirical (SADABS-2016/2)
Max./min. transmission	0.7456/0.5142
Structure solution method	Direct methods (SHELXS)
Refinement method	Full-matrix least-squares on F^2 (SHELXL-2018/3)
Data/restraints/parameters	650/0/27
Goodness-of-fit on F^2	1.096
Final R indices [$I > 2\sigma(I)$]	R_1 = 0.0164, wR_2 = 0.0397
R indices (all data)	R_1 = 0.0181, wR_2 = 0.0408
Theta range for data collection/deg	2.78 to 27.40
Largest diff. peak/hole	1.029/−0.958 e Å^{-3}
Extinction coefficient	0.00137(11)
Hydrogen atom treatment	Riding model
Weight scheme for the refinement	$w = 1/[s^2(Fo^2) + (0.0181P)^2 + 1.7360P]$<br>where $P = (Fo^2 + 2Fc^2)/3$
Index ranges	$-13 \le h \le 13$<br>$-13 \le k \le 13$<br>$-12 \le l \le 11$

Table S2. Atomic Coordinates and Equivalent Isotropic Displacement Parameters (Å^2).

**Atom**	**x**	**y**	**z**	**Occupancy**	**U_{eq}/U_{iso} (Å^2)**
Pd1	0.000000	0.000000	0.000000	1.000	0.01940(12)
Cs1	0.22663(3)	0.25891(2)	0.250000	1.000	0.02959(12)
Cl1	0.500000	0.27477(9)	0.02487(10)	1.000	0.0268(2)
Cl2	0.21952(9)	0.000000	0.000000	1.000	0.0277(2)
O1	0.000000	0.4920(5)	0.250000	1.000	0.0722(18)
H1	0.000000	0.539140	0.175870	1.000	0.087

Table S3. Anisotropic Displacement Parameters (Å^2).

Atom	U_{11}	U_{22}	U_{33}	U_{23}	U_{13}	U_{12}
Cl1	0.0261(4)	0.0211(4)	0.0334(4)	0.0011(3)	0	0
Pd1	0.0197(2)	0.0185(2)	0.0199(2)	−0.00020(12)	0	0
Cs1	0.03290(17)	0.03356(17)	0.02232(15)	0	0	−0.00765(10)
O1	0.093(5)	0.062(4)	0.062(4)	0	0	0
Cl2	0.0221(4)	0.0253(5)	0.0358(5)	0.016(3)	0	0

Table S4. Selected Bond Lengths.

Bond	Length/Å
Pd1-Cl1	2.3103(9)
Pd1-Cl2	2.3034(9)
Cs1-Cl1	3.5381(7)
Cs1-Cl1	3.5748(6)
Cs1-Cl2	3.4572(2)
Cs1-Cl2	3.54487(19)
Cs1-O1	3.363(4)
O1-H1	0.8500

Table S5. TRPL fitting parameters for room temperature and low temperature of $Cs_2PdCl_4{\cdot}H_2O$ SCs.

	**300 K**	**77 K**
Lifetime component (ns)	τ_1 = 1.53, τ_2 = 9.05	τ_1 = 2.87, τ_2 = 14.79
Amplitude proportion (%)	τ_1 = 41.01%, τ_2 = 58.99%	τ_1 = 32.87%, τ_2 = 67.13%
Average lifetime (ns)	$\langle\tau\rangle_{amp}$ = 3.01, $\langle\tau\rangle_{int}$ = 5.97	$\langle\tau\rangle_{amp}$ = 6.26, $\langle\tau\rangle_{int}$ = 10.87
Goodness of fit (χ^2)	1.286	1.190
