# Supplementary material for: Synthesis, Crystal Structure, Spectroscopic Properties, and Visible-Light Photoresponse of Lead-Free Cs2PdCl4·H2O
Source: Molecules. 2026 Sep 14;31(18):3259. doi: 10.3390/molecules31183259 (PMC13609189; doi:10.3390/molecules31183259)

checkCIF/PLATON report

You have not supplied any structure factors. As a result the full set of tests cannot be run.

THIS REPORT IS FOR GUIDANCE ONLY. IF USED AS PART OF A REVIEW PROCEDURE FOR PUBLICATION, IT SHOULD NOT REPLACE THE EXPERTISE OF AN EXPERIENCED CRYSTALLOGRAPHIC REFEREE.

No syntax errors found. CIF dictionary Interpreting this report

Datablock: 1

Bond precision: Pd-Cl = 0.0009 A Wavelength=0.71073

Cell:	a=10.4929(3)	b=10.2045(3)	c=9.4489(3)
	alpha=90	beta=90	gamma=90
Temperature:	223 K		

	Calculated	Reported
Volume	1011.74(5)	1011.74(5)
Space group	C m c m	C m c m
Hall group	-C 2c 2	-C 2c 2
Moiety formula	Cl8 Pd2, 2(H2 O), 4(Cs)	Cl1.6 Cs0.8 H0.8 O0.4 Pd0.4
Sum formula	Cl8 Cs4 H4 O2 Pd2	Cl1.60 H0.80 Cs0.80 O0.40 Pd0.40
Mr	1064.07	212.81
Dx,g cm-3	3.493	3.493
Z	2	10
Mu (mm-1)	9.910	9.910
F000	936.0	936.0
F000'	931.69	
h,k,lmax	13,13,12	13,13,12
Nref	652	650
Tmin,Tmax		0.514,0.746
Tmin'		

Correction method= # Reported T Limits: Tmin=0.514 Tmax=0.746
AbsCorr = NONE

Data completeness= 0.997 Theta(max)= 27.401

R(reflections)= 0.0164(599) wR2(reflections)= 0.0408(650)

S = 1.096 Npar= 27

The following ALERTS were generated. Each ALERT has the format
test-name_ALERT_alert-type_alert-level.
Click on the hyperlinks for more details of the test.

Alert level C

```
CRYSC01_ALERT_1_C The word below has not been recognised as a standard
            identifier.
            yellowish
PLAT041_ALERT_1_C Calc. and Reported SumFormula    Strings  Differ     Please Check
              Calc: Cl1.60 Cs0.80 H0.80 O0.40 Pd0.40
              Rep.: Cl1.60 H0.80 Cs0.80 O0.40 Pd0.40
PLAT042_ALERT_1_C Calc. and Reported MoietyFormula Strings  Differ     Please Check
              Calc: Cl8 Pd2, 2(H2 O), 4(Cs)
              Rep.: Cl1.6 Cs0.8 H0.8 O0.4 Pd0.4
PLAT053_ALERT_1_C Minimum Crystal Dimension Missing (or Error) ...     Please Check
PLAT054_ALERT_1_C Medium  Crystal Dimension Missing (or Error) ...     Please Check
PLAT055_ALERT_1_C Maximum Crystal Dimension Missing (or Error) ...     Please Check
```

Alert level G

```
PLAT004_ALERT_5_G Polymeric Structure Found with Maximum Dimension          2 Info
PLAT007_ALERT_5_G Number of Unrefined Donor-H Atoms .............          1 Report
              H1
PLAT012_ALERT_1_G No       _shelx_res_checksum Found in CIF ......     Please Check
PLAT045_ALERT_1_G Calculated and Reported Z Differ by a Factor ...      0.200 Check
PLAT794_ALERT_5_G Tentative Bond Valency for Pd1       (II)      .       2.00 Info
```

0 **ALERT level A** = Most likely a serious problem - resolve or explain
0 **ALERT level B** = A potentially serious problem, consider carefully
6 **ALERT level C** = Check. Ensure it is not caused by an omission or oversight
5 **ALERT level G** = General information/check it is not something unexpected

8 ALERT type 1 CIF construction/syntax error, inconsistent or missing data
0 ALERT type 2 Indicator that the structure model may be wrong or deficient
0 ALERT type 3 Indicator that the structure quality may be low
0 ALERT type 4 Improvement, methodology, query or suggestion
3 ALERT type 5 Informative message, check

It is advisable to attempt to resolve as many as possible of the alerts in all categories. Often the minor alerts point to easily fixed oversights, errors and omissions in your CIF or refinement strategy, so attention to these fine details can be worthwhile. In order to resolve some of the more serious problems it may be necessary to carry out additional measurements or structure refinements. However, the purpose of your study may justify the reported deviations and the more serious of these should normally be commented upon in the discussion or experimental section of a paper or in the "special_details" fields of the CIF. checkCIF was carefully designed to identify outliers and unusual parameters, but every test has its limitations and alerts that are not important in a particular case may appear. Conversely, the absence of alerts does not guarantee there are no aspects of the results needing attention. It is up to the individual to critically assess their own results and, if necessary, seek expert advice.

Publication of your CIF in IUCr journals

A basic structural check has been run on your CIF. These basic checks will be run on all CIFs submitted for publication in IUCr journals (*Acta Crystallographica*, *Journal of Applied Crystallography*, *Journal of Synchrotron Radiation*); however, if you intend to submit to *Acta Crystallographica Section C* or *E* or *IUCrData*, you should make sure that full publication checks are run on the final version of your CIF prior to submission.

Publication of your CIF in other journals

Please refer to the *Notes for Authors* of the relevant journal for any special instructions relating to CIF submission.

PLATON version of 22/08/2024; check.def file version of 21/08/2024

Datablock 1 - ellipsoid plot

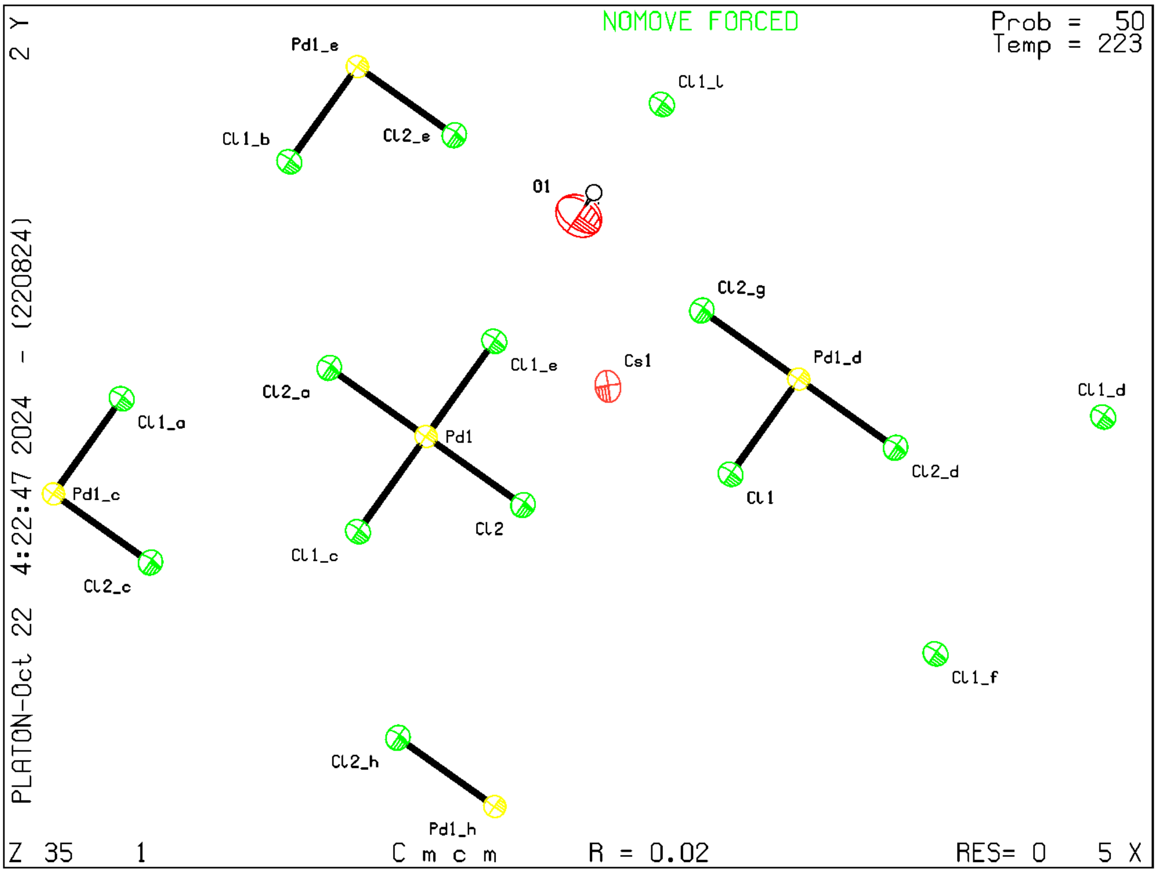

Supplement: Supplementary file 1 [file molecules-31-03259-s001.zip › Cs2PdCl4·H2O checkcif.pdf]
